# Urinary pro-thrombotic, anti-thrombotic, and fibrinolytic molecules as biomarkers of lupus nephritis

**DOI:** 10.1186/s13075-019-1959-y

**Published:** 2019-07-18

**Authors:** Ling Qin, Samantha Stanley, Huihua Ding, Ting Zhang, Van Truong, Teja Celhar, Anna-Marie Fairhurst, Claudia Pedroza, Michelle Petri, Ramesh Saxena, Chandra Mohan

**Affiliations:** 1 Department of Nephrology & Rheumatology, Shanghai Tenth People’s Hospital, Tongji University School of Medicine, Shanghai, People’s Republic of China; 2 Department of Biomedical Engineering, University of Houston, 3605 Cullen Boulevard, Houston, TX 77204 USA; 3 Department of Pediatrics, UT Houston, Houston, TX USA; 4 Singapore Immunology Network, Agency for Science, Technology, and Research, Singapore, Singapore; 5 Department of Rheumatology, John Hopkins Medical University, Baltimore, MD USA; 6 Department of Nephrology, UT Southwestern Medical Center, Dallas, TX USA

**Keywords:** Lupus nephritis, Biomarkers, Plasmin, Tissue factor, Tissue factor pathway inhibitor, D-dimer

## Abstract

**Objective:**

This study evaluates the utility of urinary pro-thrombotic molecules such as tissue factor (TF), anti-thrombotic molecules such as tissue factor pathway inhibitor (TFPI), and fibrinolytic molecules such as plasmin and d-dimer as biomarkers of lupus nephritis (LN).

**Methods:**

Urine samples from 113 biopsy-proven LN patients (89 active LN and 24 inactive LN), 45 chronic kidney disease patients, and 41 healthy controls were examined for d-dimer, plasmin, TF, and TFPI levels by ELISA. The area under the receiver operating characteristic curve (AUC) analysis, multivariate regression analysis, and Bayesian network analysis were performed to assess the diagnostic value of the assayed molecules in LN.

**Results:**

Although urinary d-dimer, plasmin, TF, and TFPI were all elevated in active LN compared to all control groups, and correlated with rSLEDAI and SLICC RAS disease activity indices, urine plasmin emerged as the strongest independent predictor of eGFR and renal disease status, by multivariate regression analysis and Bayesian network analysis. Whereas urine plasmin discriminated active LN from inactive disease with an AUC of 0.84, the combination of urine plasmin and TFPI discriminated ALN from ILN with an AUC of 0.86, with both surpassing the specificity and positive predictive value of traditional markers such as anti-dsDNA and complement C3.

**Conclusion:**

Both thrombogenic and thrombolytic cascades appear to be upregulated in lupus nephritis, with proteins from both cascades appearing in the urine. Of the coagulation cascade proteins surveyed, urine plasmin emerges as the strongest predictor of eGFR and clinical renal disease in patients with LN.

**Electronic supplementary material:**

The online version of this article (10.1186/s13075-019-1959-y) contains supplementary material, which is available to authorized users.

## Introduction

Systemic lupus erythematosus (SLE) is a systemic autoimmune disease with multiple organ involvement, characterized by diverse autoantibody production, notably anti-DNA and anti-nuclear antibodies. Lupus nephritis (LN) is one of the most frequent and severe clinical manifestations of SLE, representing a leading cause of morbidity and mortality. Although novel immunosuppressive drugs and biologics therapy have brought improvements in recent SLE/LN survival rates, early diagnosis and monitoring disease flares are still challenges that need to be addressed. Current laboratory parameters, including anti-dsDNA, C3, proteinuria, and eGFR, are not reliable for early diagnosis and monitoring of treatment responses, and a renal biopsy remains the gold standard for the diagnosis and prognosis of LN. However, this procedure is invasive and cannot be used for routine monitoring of disease activity and treatment responses. Because of this, several studies focusing on screening and identifying non-invasive biomarkers for the early diagnosis and monitoring of SLE and LN are emerging [1]. Urine is easily collected and may reflect the underlying renal inflammation and injury more accurately than serum. Therefore, urine biomarkers represent promising candidates for the early diagnosis as well as the monitoring of disease activity and therapeutic responses in LN.

It has been reported that lupus nephritis is associated with hypercoagulability [2]. Coagulation system disorders have been reported in lupus nephritis patients [3] and murine lupus nephritis [4]. The frequency of thrombotic events was documented to be higher in SLE patients than in the general population, and these events were associated with poor outcome [5]. More interestingly, significantly increased intra-renal microthrombosis has been reported in lupus nephritis, associated with more severe renal pathology and clinical disease [6–9]. If this is the case, we wondered if proteins from the coagulation cascade might be elevated in the urine of LN patients. This study was designed to explore if urinary proteins related to coagulation (namely tissue factor, TF, and tissue factor pathway inhibitor, TFPI) or clot lysis (namely plasmin and D-dimer) were elevated in LN, and if so, whether they can function as disease biomarkers.

## Patients and methods

### Patients

Samples for this study were obtained from patients with LN and controls who had previously been recruited from the renal clinic at UT Southwestern Medical Center (UTSW) between 2007 and 2011. Urine samples as well as clinical data were collected at the time of patient visit. Totally, 113 biopsy-proven LN patients (89 active LN and 24 inactive LN) were enrolled. Forty-five gender and age-matched patients with chronic kidney disease (CKD) and 41 healthy volunteers were recruited as disease controls and healthy controls respectively. Patient characteristics and medication history can be found in Table 1. All SLE patients satisfied the ACR criteria for SLE [10]. Disease activity was assessed using SLEDAI (SLE disease activity index) [11], renal SLEDAI (rSLEDAI) [12], and SLICC RAS (The Systemic Lupus International Collaborating Clinics Renal Activity Score) [13]. Clinical data was gathered by chart review, and SLEDAI was calculated based on chart review. SLE patients were then classified as having either active LN (ALN) or inactive LN (ILN). Active LN was defined as active urine sediment or proteinuria (rSLEDAI > 0). Inactive LN was defined as inactive urine sediment and no proteinuria (rSLEDAI = 0). The study was approved by the Medical Ethics Committee of the Hospital, and informed consent was obtained from all participants following the declaration of the Convention of Helsinki.

**Table 1:** Demographics and clinical characteristics of LN patients

	ALN (*N* = 89)	ILN (*N* = 24)	CKD (*N* = 45)	HC (*N* = 41)
Age* (years)	33.4 ± 10.1	36.6 ± 12.5	48.3 ± 12.5	32.9 ± 7.9
Female, no. (%)	73 (82)	20 (83)	15 (34)	22 (56)
Asian/African American/Hispanic/Caucasia, no.	3/35/37/12	3/33/37/12	2/11/14/17	3/10/14/11
SLEDAI**	10 (6–18)	2 (0–4)	N/A	N/A
rSLEDAI**	8 (4–12)	0 (0–0)	N/A	N/A
Protein-to-creatinine ratio** (mg/mg)	1.73 (0.9–3.4)	0 (0.1–0.2)	1.04 (0.203–2.44)	
eGFR** (mL/min/1.73 m^2^)	64 (34.75–111.5)	69 (27–102.75)	56.5 (34.3–84.5)	
Positive ANA/total tested	38/83	11/24	N/A	N/A
Positive anti-dsDNA/total tested	34/89	8/24	N/A	N/A
Hypocomplementemia/total tested	50/89	9/24	N/A	N/A
Renal pathology, no. (%)	I: 0 (0)	I: 0 (0)	DN: 15 (35)	
	II: 6 (7)	II: 4 (17)	FSGS: 6 (14)	
	III/ III+V: 22 (25)	III/III+V: 7 (29)	MN: 5 (11)	
	IV/IV+V: 41 (46)	IV/IV+V: 8 (33)	MN+FSGS: 1 (2)	
	V: 14 (16)	V: 3 (13)	MCD: 3 (7)	
	VI: 1 (1)	VI: 0 (0)	ANCA-GN: 6 (13)	
	Unknown: 5 (6)	Unknown: 2 (8)	Crescent GN: 1 (2)	
			BANS: 1 (2)	
Comorbidity, no. (%)				
Hypertension	39 (46)	8 (33)	34 (77)	
Diabetes mellitus	4 (5)	1 (4)	18 (41)	
Hyperlipidemia	36 (40)	4 (17)	26 (60)	
Hypothyroidism	6 (7)	1 (4)	2 (5)	
Pulmonary embolism	7 (8)	2 (8)	3 (7)	
Current medications, no. (%)				
Prednisone	65 (73)	14 (58)	12 (28)	
Cyclophosphamide	9 (10)	0 (0)	1 (2)	
Mycophenolate mofetil	23 (26)	10 (42)	4 (9)	
Azathioprine	6 (7)	4 (17)	0 (0)	
Methotrexate	1 (1)	1 (4)	1 (2)	
Cyclosporine/tacrolimus	1 (1)	2 (8)	1 (2)	
Hydroxychloroquine	43 (48)	17 (71)	1 (2)	
ACE inhibitors/ARB	45 (51)	14 (58)	30 (70)	
Anti-coagulation/platelets	2 (2)	3 (13)	N/A	

*LN* lupus nephritis, *CKD* chronic kidney disease, *HC* healthy control, *SLEDAI* systemic lupus erythematosus disease activity index, *rSLEDAI* renal SLEDAI, *eGFR* estimated glomerular filtration rate, *ANA* antinuclear antibodies, *dsDNA* double-stranded DNA, *ACE* angiotensin-converting enzyme, *ARB* angiotensin receptor blocker, *DN* diabetic nephropathy, *FSGS* focal segmental glomerulosclerosis, *MN* membranous nephropathy, *MCD* minimal change disease, *ANCA-GN* anti-neutrophil cytoplasmic antibody-associated glomerulonephritis, *BANS* benign arteriolar nephrosclerosis

*Mean ± standard error of the mean

**Median (Q1–Q3)

### ELISA

Urinary levels of d-dimer, plasmin, TF, and TFPI were determined using human ELISA kits from Raybiotech (Norcross, GA, USA), Lifespan Biosciences (Seattle, WA, USA), R&D Systems (Minneapolis, MN, USA), and R&D Systems (Minneapolis, MN, USA) respectively, according to the manufacturer’s instructions. Briefly, diluted urine samples were added in pre-coated 96-well microplates. After sample incubation, detection antibodies were added, followed by streptavidin-HRP, and substrate. A microplate reader (ELX808 from BioTek Instruments, Winooski, VT) was used to read the optical density at 450 nm. Urine samples were diluted 1:2000, 1:100, 1:4, and 1:2 for d-dimer, plasmin, TF, and TFPI, respectively. The optimal concentration was determined based on a standard curve derived for each molecule.

### Urine creatinine assay and renal function assessment

Urinary creatinine concentrations were determined using Creatinine Parameter Assay Kit (R&D Systems, Minneapolis, MN). Urine creatinine concentrations were used to account for the glomerular filtration rate and hydration status of the patient; each protein concentration was divided by the urinary creatinine concentration to normalize the proteins to the levels of urinary creatinine. Estimated glomerular filtration rate (eGFR) was calculated using the MDRD Study equation for renal function assessment [14].

### Statistics

Data were analyzed and plotted using GraphPad Prism 5 and Matlab (R2015a). Kolmogorov-Smirnov test was used to assess the normality of the data. For comparisons of multiple groups, ANOVA test and subsequent post-test pairwise comparisons were used. For correlation analysis, the Pearson method or the nonparametric Spearman method was used. Linear regression, LASSO regression, and receiver operating characteristic (ROC) curve were used to assess the performance of urine biomarkers in distinguishing ALN patients from ILN patients, CKD patients, and healthy subjects. A two-tail *p* value less than 0.05 was considered significant.

### Diagnostic performance of novel urine markers and comparison to conventional markers

Once the urine biomarker concentrations were normalized to urinary creatinine, any values below the limit of detection were replaced with 10% of the lowest detected value for that protein, and these values were then log-transformed and sorted based on their disease status for model construction. Once the data was log transformed, each sample was assigned a random score between 0 and 1000 using Excel’s random number generator, and the samples were sorted by these scores. The samples were then split into 2 equal-sized groups: one group would be used for model construction, while the other was used for model validation. The model development groups consisted of 44 active lupus nephritis and either 12 inactive lupus nephritis or 20 healthy controls, while the model validation groups contained the remaining 44 active lupus nephritis and either 12 inactive lupus nephritis or 20 healthy controls. The group for model construction was then imported into Matlab and used for LASSO regression analysis to determine which panel of 2, 3, or 4 biomarkers was most efficient at discriminating active lupus nephritis.

### Bayesian network (BN) analysis

BN analysis was performed using the BayesiaLab software (Bayesia, version 7.0.1) [15]. The dataset for unsupervised learning included 78 patients with active LN and 22 patients with inactive LN with the following parameters: new urinary biomarkers (TFPI, D-dimer, plasmin, TF), demographic data (age, race, sex), and disease measures (glomerulonephritis class, AI, CI, rSLEDAI, SLICC, and eGFR). Only patients with a complete dataset (no missing values) were included in the analysis. Continuous data were discretized into 3 bins using the R2-GenOpt algorithm, and the EQ algorithm with structural coefficient (*α*) 0.4 was used for unsupervised learning of the network [15]. Under these conditions, all parameters except race were connected in the generated model.

## Results

### Study population

Samples from 113 renal biopsy-proven LN patients were included in this study. The patients were divided into active LN (ALN, active urine sediment or proteinuria, rSLEDAI > 0) and inactive LN (ILN, inactive urine sediment and no proteinuria, rSLEDAI = 0). The demographics and clinical characteristics of the LN patients and controls are shown in Table 1; information about patient comorbidity and medication history are also listed. The mean ± SD ages of active LN patients and inactive LN patients were 33.4 ± 10.1 and 36.6 ± 12.5 years, respectively. The mean SLEDAI and rSLEDAI scores were 12, 8 for ALN and 2, 0 for ILN patients, respectively. Samples from 45 CKD patients and 41 healthy controls were also included in this study.

### Levels of urinary protein markers in LN patients and controls

**Fig. 1:**
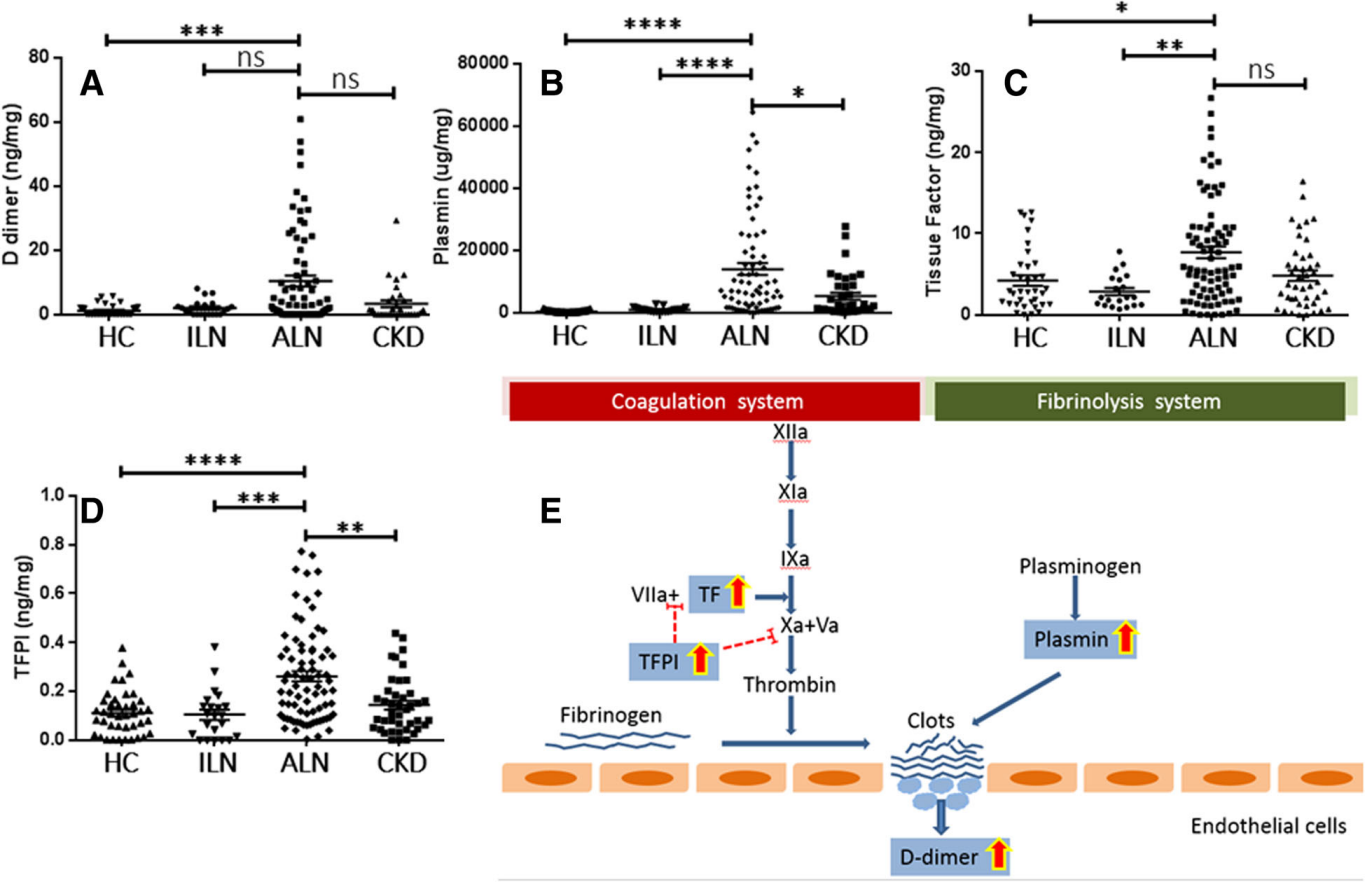
Urinary pro-thrombotic, anti-thrombotic, and fibrinolytic molecules are significantly elevated in active lupus nephritis. Plotted are urine concentrations of d-dimer (**a**), plasmin (**b**), TF (**c**), and TFPI (**d**), as determined by ELISA in active LN (*N* = 89), inactive LN (*N* = 24), CKD patients (*N* = 45), and healthy controls (*N* = 41) after normalization by urinary creatinine. All four molecules were significantly elevated in ALN patients compared to ILN patients and healthy controls. Each dot represents an individual subject. **e** The function of the four assayed biomarkers within the coagulation and fibrinolysis systems; red broken line indicates an inhibitory role, while a blue arrow indicates an activation role. The urine biomarkers interrogated in this study all play key roles in the depicted pro-/anti-coagulation and/or fibrinolysis systems

The urinary levels of the four selected proteins in the different groups are compared in Fig. 1a–d and Additional file 1: Table S1. ALN patients showed higher urinary levels of plasmin (*p* < 0.0001), TF (*p* < 0.01), and TFPI (*p* < 0.001) compared to the ILN patients. When compared to the CKD patients, the urine levels of plasmin and TFPI of ALN patients were also significantly increased (*p* < 0.01, *p* < 0.05, respectively). The urinary levels of d-dimer, plasmin, TF, and TFPI were all significantly elevated in ALN patients compared to healthy controls (*p* < 0.001, *p* < 0.0001, *p* < 0.05, *p* < 0.0001, respectively). The inter-relationships between the 4 assayed molecules are displayed in Fig. 1e.

### Correlation analysis between novel protein markers and clinical parameters

**Fig. 2:**
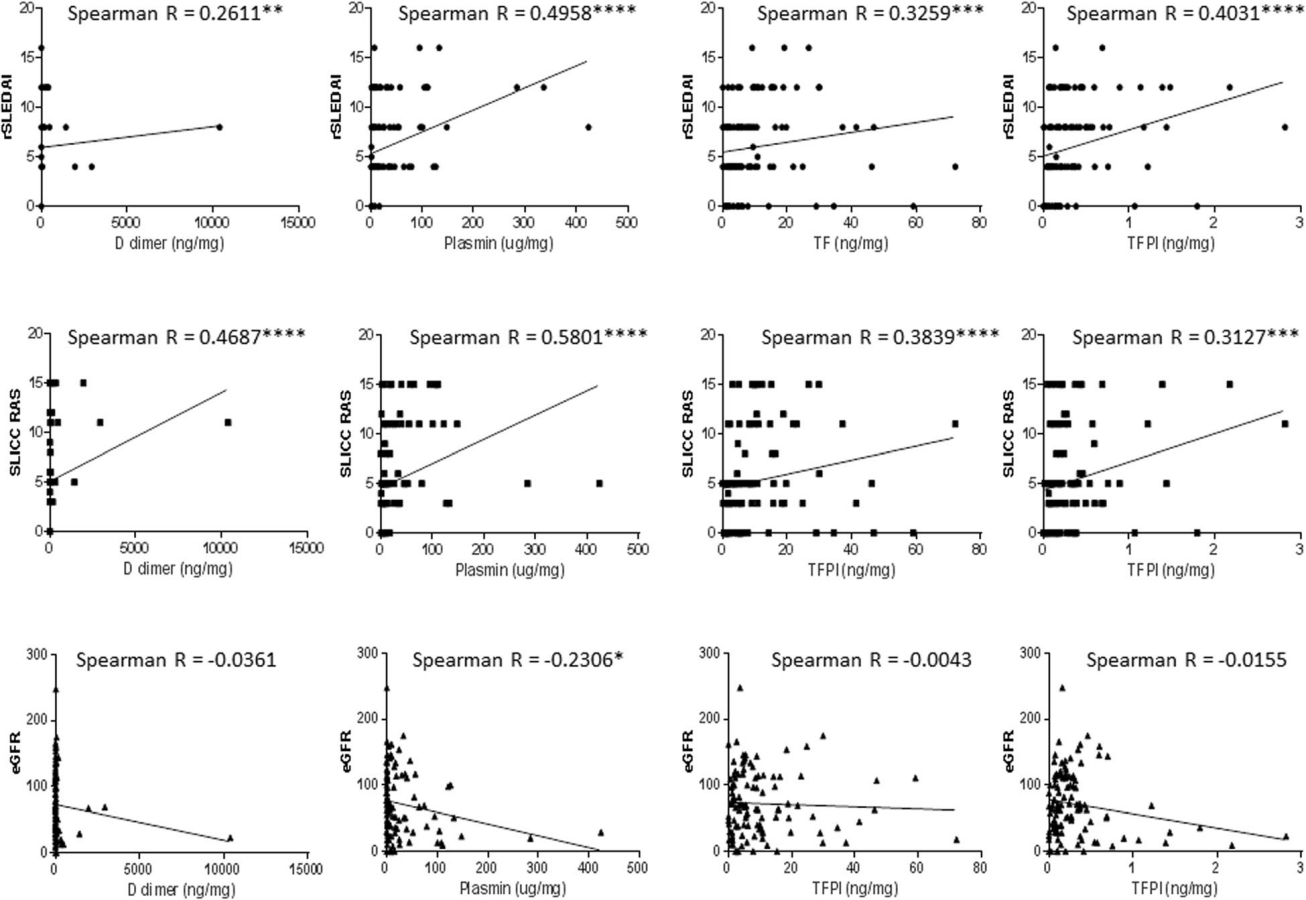
Correlation between urine biomarkers and clinical parameters in LN patients. Each plot indicates the correlation patterns of urinary creatinine-normalized levels of d-dimer, plasmin, TF, and TFPI against the following parameters: rSLEDAI (row 1), SLICC RAS (row 2), and eGFR (row 3). The same urine biomarker data plotted in Fig. 1 were used to generate these correlation plots

As demonstrated in Fig. 2, urinary levels of d-dimer, plasmin, TF, and TFPI correlated positively with rSLEDAI (*r* = 0.26 *p* < 0.01, *r* = 0.50 *p* < 0.0001, *r* = 0.33 *p* < 0.0001, *r* = 0.40 *p* < 0.0001, respectively) and SLICC RAS (*r* = 0.47 *p* < 0.0001, *r* = 0.58 *p* < 0.0001, *r* = 0.40 *p* < 0.0001, *r* = 0.31 *p* < 0.001, respectively). Plasmin also showed a weak but statistically significant negative correlation with eGFR (r = − 0.23, *p* < 0.05), as shown in Fig. 2, meaning that as urine plasmin increased, renal function, as gauged by eGFR, worsened.

### Diagnostic performance of novel urine markers in comparison to conventional markers

**Fig. 3:**
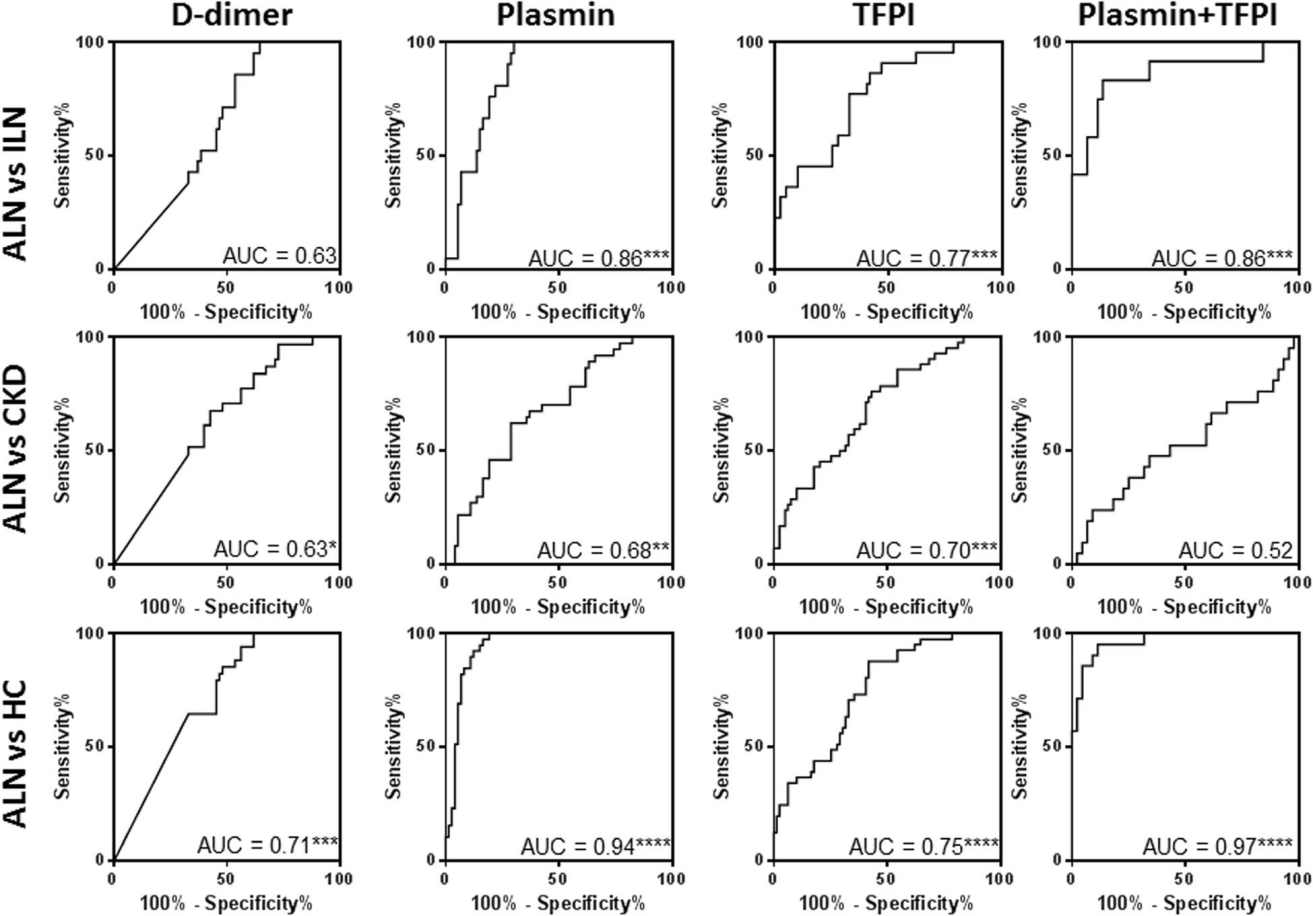
Performance of urine biomarkers in discriminating ALN patients from ILN patients, CKD patients, and healthy controls. The same urine biomarker data plotted in Fig. 1 were used to generate these plots. The area under the curve (AUC) is shown within each graph, with the following legend describing the statistical significance: ****p < 0.0001, ***p < 0.001, **p < 0.01, *p < 0.05. Urinary creatinine-normalized plasmin and TFPI were both able to successfully differentiate ALN patients from ILN patients (row 1) and healthy controls (row 3). Plasmin, TF, and TFPI successfully discriminated ALN patients from the CKD controls (row 2). The combination of plasmin and TFPI improved the discriminatory potential (last column)

Plasmin, TFPI, and TF individually performed well in distinguishing ALN from ILN (AUC = 0.86 *p* < 0.0001, AUC = 0.77 *p* < 0.0001, AUC = 0.74 *p* < 0.0001, respectively, as displayed in Fig. 3). D-dimer, plasmin, TFPI, and TF all performed well in distinguishing ALN from healthy controls (AUC = 0.71 *p* < 0.001, AUC = 0.94 *p* < 0.0001, AUC = 0.75 *p* < 0.0001, AUC = 0.66 *p* < 0.01, respectively, as is also shown in Fig. 3), as well as from CKD patients (AUC = 0.63 *p* < 0.05, AUC = 0.68 *p* < 0.01, AUC = 0.70 *p* < 0.001, AUC = 0.62, *p* < 0.05, as shown in Fig. 3). The performance of these urine biomarkers is compared to that of anti-dsDNA and C3/C4 in Table 2. It can be seen that both plasmin and D-dimer showed 100% sensitivity in distinguishing ALN from ILN. D-dimer, plasmin, TFPI, and TF all performed better in sensitivity and positive predictive values (PPV) (sensitivity = 100%, PPV = 93.2%; sensitivity = 100%, PPV = 95.7%; sensitivity = 60.5%, PPV = 88.9%; sensitivity = 86.4%, PPV = 91.5%) than anti-ds DNA (sensitivity = 40.0%, PPV = 84.9%) and C3/C4 (sensitivity = 56.3%, PPV = 82.1%). Plasmin and TF performed better in terms of specificity and negative predictive values (NPV) (specificity = 69.9%, NPV = 50.0%; specificity = 85.0%, NPV = 34.7%) than anti-ds DNA (specificity = 66.7%, NPV = 22.6%) and C3/C4 (specificity = 61.9%, NPV = 27.1%) in discriminating ALN from ILN.

**Table 2:** Diagnostic performance of urine biomarkers in differentiating active LN from inactive LN compared to conventional markers

ALN vs ILN Linear regression	AUC	Cutoff	Sensitivity (%)	Specificity (%)	PPV (%)	NPV (%)
D-dimer	0.63	8.15	100	35.6	93.2	29.4
Plasmin	0.86***	2875	100	69.9	95.7	50.0
TF	0.74***	4.86	60.5	85.0	88.9	34.7
TFPI	0.77***	0.19	86.4	58.2	91.5	35.8
Positive anti-dsDNA			40.0	66.7	84.9	22.6
Low complement			56.3	61.9	82.1	27.1
LASSO regression						
Plasmin+TFPI	0.86***	0.76	83.3	86.4	95.0	63.2

***p < 0.001

Next, we asked if combining 2, 3, or 4 biomarkers had better potential to discriminate ALN from the controls. Of all possible combinations tried, a biomarker panel comprised of plasmin and TFPI performed most effectively in discriminating ALN from HC, with an improved AUC value of 0.97 (*p* < 0.0001). Furthermore, the combination of urine plasmin and TFPI showed higher specificity and negative predictive values than urine plasmin (86.4% vs 69.9%; 63.2% vs 50.0%) when compared to anti-dsDNA and complement C3. However, none of the multi-marker panels performed better than plasmin in distinguishing ALN from ILN, as is evident from Fig. 3.

### Univariate and multivariate regression analysis for confounding factors

In multivariate regression analysis adjusting for age, ethnicity, and gender, plasmin (*p* < 0.016) and TFPI (*p* < 0.027) were the only independent predictors of eGFR among the 4 biomarkers tested, with plasmin being the strongest (Additional file 1: Table S2). Age was also an independent predictor of eGFR (*p* < 0.013), as expected. Similarly, plasmin and TFPI were once again the only independent predictors of SLEDAI, besides the female gender (Additional file 1: Table S2). In univariate analysis of biomarkers in relation to drug usage (prednisone, MMF, or plaquenil), urine TF was the only marker that showed any significant association—urine TF was significantly higher in patients taking ≥ 10 mg/day prednisone (*p* < 0.027).

### Network analysis reveals plasmin to be a major driver of disease

**Fig. 4:**
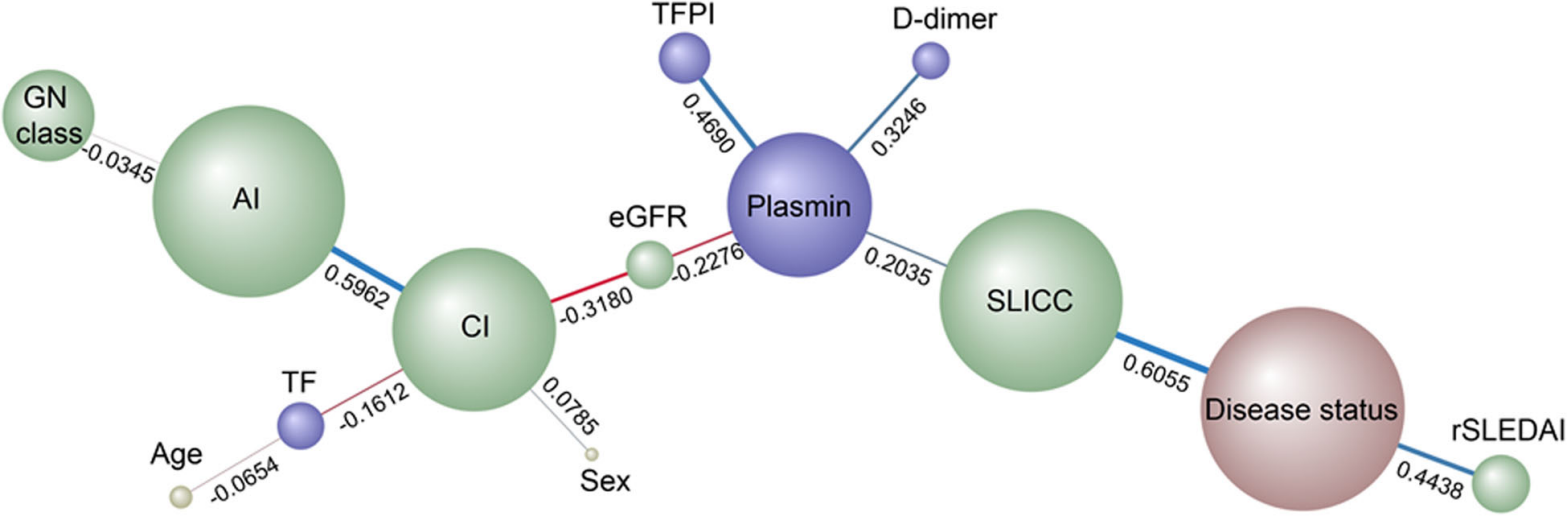
Bayesian network analysis of urine biomarker levels in relation to clinical and pathological indices in a cohort of LN patients. The same urine biomarker data plotted in Fig. 1, and the clinical features of the study subjects were subjected to Bayesian network analysis using BayesiaLab. The network shown was constructed in an unsupervised manner, using the EQ algorithm and a structural coefficient of 0.4. The circular nodes that make up the Bayesian Network represent the variables of interest, including urine biomarkers (purple-colored), histological or clinical indices (green-colored), demographic data (yellow-colored), and disease status (active LN versus inactive disease versus no disease) (colored brown). The size of each node denotes the “node force,” which is related to its impact on other nodes in the network, based on conditional probabilities. The links (arcs) that interconnect the nodes represent informational or causal dependencies among the variables, including the correlation coefficients between neighboring nodes, as listed. Blue and red links represent positive and negative correlation, respectively, with the thickness of the link being proportional to the correlation coefficient

Bayesian network analysis uses probability distributions to represent all changing variables in a model and how they relate to each other [16]. Directed acyclic graphs that represent such probabilistic models called Bayesian networks [15, 16] are particularly apt when faced with the “curse of dimensionality,” i.e., when the number of predictors is very high. We subjected the quantities of the 4 assayed markers and various clinical metrics to unsupervised Bayesian network analysis. As shown in Fig. 4, and as expected, the 3 clinical indices of renal disease, SLICC, disease status (active lupus nephritis versus inactive lupus), and rSLEDAI, were strongly linked to each other, with strong positive correlation. The fact that this “ground truth” relationship among these 3 quantities was correctly identified by the unsupervised Bayesian network algorithm offers internal validation of this approach.

More importantly, plasmin emerged as a major driver of variations (across the dataset) in all 3 of the clinical indices described above, eGFR and renal pathology chronicity index, as well as the biomarkers, d-dimer, and TFPI. eGFR was negatively correlated with both urine plasmin and chronicity index (Fig. 4). The latter relationship has already been established in the literature, again offering internal validation of the Bayesian algorithm adopted. More interestingly, both urine plasmin and renal pathology chronicity index were equal-potent in dictating eGFR, as evidenced by their similar impact force (which is proportional to the size of each node) as well as the strength of negative correlation with eGFR (Fig. 4). The relative impact of the other 3 biomarker proteins, TFPI, d-dimer, and TF, on clinical indices or renal pathology indices was modest, compared to that of urine plasmin.

## Discussion

In this cross-sectional study, we assessed the performance of four urine biomarker candidates that are involved in coagulation or fibrinolysis. Our data indicates that urinary levels of d-dimer, plasmin, TF, and TFPI are all elevated in active LN patients compared to inactive LN patients and healthy controls. All four proteins correlated with systemic disease activity and renal disease activity. Importantly, urine plasmin performed best among the four proteins in discriminating active LN from inactive disease, even better than traditional markers, such as anti-dsDNA and complement C3. Furthermore, the combination of urine plasmin and TFPI showed higher specificity and negative predictive values than urine plasmin when compared to anti-dsDNA and complement C3. These elevations did not appear to be related to anti-phospholipid syndrome, as only 3 patients in this cohort had significantly elevated antibodies to cardiolipin and beta2GPI. Likewise, there was no association with any potential medications, as only 2 of the 89 patients with active LN were on anti-platelet medications (Table 1).

Of the 4 proteins assayed, urine plasmin clearly outperformed the rest as evidenced by the following: (a) it showed the strongest positive correlation with SLICC and renal SLEDAI and the strongest negative correlation with eGFR; (b) it offered the best discriminatory potential in distinguishing patients with active renal disease from the rest, with the highest AUC values; (c) in multivariate analysis, urine plasmin emerged as the strongest independent predictor of eGFR, after adjusting for age, gender, and ethnicity; and (d) by unsupervised Bayesian network analysis, plasmin again emerged as the variable with the strongest impact on clinical indices and eGFR.

Bayesian network analysis has been used to identify diagnostic and prognostic markers. It can describe the mutual relationships among biological variables and identify key driver(s) in complex biological networks [17, 18]. The network is generated using combined conditional probabilities of each node (or variable) affecting all other nodes in the network. Urine plasmin emerged as a major driver of variations in the Bayesian network that was constructed in this study. Interestingly, urine plasmin exhibited similar impact force as the renal pathology chronicity index in dictating eGFR, in the constructed Bayesian network. This conclusion is consistent with the traditional multivariate regression analysis.

Released by activated plasminogen, plasmin is a fibrinolytic serine protease that can break down blood clots into fibrin degradation products (FDP) including d-dimer. One important question relates to the likely origin of plasmin in the urine of LN patients—is it serum derived or of renal origin? Studies focusing on circulating plasminogen/plasmin levels in SLE patients reported contradicting results; some studies have found increased plasminogen/plasmin levels compared to healthy controls [19, 20], while others have seen no change in serum levels of plasminogen/plasmin in SLE [21, 22]. Indeed, we assayed serum plasmin in the same subjects included in this study. As shown in Additional file 1: Figure S1, serum plasmin did not differ significantly between the study groups, and there was no correlation between serum plasmin and urine plasmin in our data set.

Impaired systemic fibrinolysis and hypercoagulability have been implicated as a risk factor for cardiovascular diseases in SLE patients [23]. Based on literature reports and our own finding (Additional file 1: Figure S1), there is little evidence to suggest that the elevated urine plasmin in LN is of systemic origin. Although we have not studied the expression of plasmin within the kidneys, we have previously reported that the autocatalytic product of plasmin, namely angiostatin, is elevated in expression within the kidneys in LN patients [24]. Indeed, in that study, we had noted that urine angiostatin correlated with the renal pathology chronicity index, which resonates well with the relationship between urine plasmin and the chronicity index that is predicted by Bayesian analysis in this study. In support of the hypothesis that urine plasmin in LN is largely of renal origin are murine studies which reported that plasminogen can be activated by tubular urokinase-type plasminogen activator and converted to plasmin in nephrotic urine. [25] In that study, the conversion of plasminogen to plasmin occurred after glomerular filtration, suggesting that urine plasmin was not solely the product of glomerular filtration of blood [25].

A more challenging question pertains to whether elevated plasmin in LN is pathogenic or protective. In this regard, the plasminogen/plasmin system has been demonstrated to play a protective role in crescentic nephritis in animal models [26]. In another study, plasmin was shown not to be protective and may actually play a pathogenic role in experimental renal interstitial fibrosis [27]. The contribution of plasmin appears to be context-dependent and may vary with the thrombogenic state of the organism; hence, this needs to be further examined in murine models of lupus nephritis.

TF and TFPI are two key but opposing mediators in the extrinsic pathway of blood coagulation. When vessel injury occurs, TF complexes with activated factor VII (FVIIa) and initiates the coagulation cascade, while TFPI inhibits the TF-FVIIa complex in an FXa-dependent manner. Next to plasmin, urine TFPI emerged as the only other independent predictor of eGFR and renal disease status in our study. Indeed, it was the only urine marker that could further enhance discriminatory potential when added to urine plasmin, in distinguishing active LN. Studies examining the circulating levels of TFPI in SLE patients have yielded contradicting conclusions. Some studies showed that plasma TFPI concentration and activity were lower in SLE patients compared to healthy controls [28–30], while others have found elevated free TFPI levels that correlated with lupus disease activity and endothelial damage [31]. Thus far, no study has measured urinary TFPI levels in SLE.

Literature is fairly consistent in suggesting that TFPI is produced within the kidneys, where it may play a protective role. TFPI has been confirmed to be secreted by human mesangial cells, podocytes, and proximal tubule cells in culture [32–34]. TFPI was also found to be induced to inhibit TF activity and reduce fibrin deposition in the chronic stages of crescentic glomerulonephritis (GN) [35]. Studies have shown that the functional inhibition of TFPI by anti-TFPI antibody can aggravate renal impairment, whereas infusion of recombinant TFPI reduced fibrin deposition, decreased levels of proteinuria and renal injury in experimental crescentic GN [36]. Taken together, it appears likely that TFPI may have been induced within the kidneys in LN (in an “attempt” to counteract increasing thrombogenesis), although this conjecture needs to be formally demonstrated.

In this study, both urinary TF and urinary TFPI were correlated strongly with one another and are both likely to be of renal origin, extrapolating from literature reports. It has been reported that urinary TF is secreted by renal tubules in normal human renal tissue, not passively filtered by the glomeruli [37]. It has been suggested that activated resident kidney cells and infiltrating inflammatory cells induced increased urinary TF expression [38]. Urinary TF levels were increased in non-crescentic GN patients compared to normal controls and were directly associated with creatinine clearance [39]. Although TF may be generated within the kidneys and also functions well in discriminating ALN patients from all controls, it is clearly outperformed by urine plasmin and TFPI in terms of diagnostic metrics.

Compared to the other 3 molecules examined, d-dimer has been well studied by several other groups. Both blood and urine d-dimer levels have been documented to be elevated in LN and other CKD, with good predictive potential for renal disease [2, 40–43]. It has also been suggested that urinary d-dimer may reflect intra-glomerular coagulation and fibrinolysis [44]. Despite all of these reports, urine d-dimer was clearly outperformed by other urinary markers in this study, notably urine plasmin and TFPI, in discriminating renal disease in SLE.

## Conclusions

In summary, this is the first systematic study to assess urinary pro-thrombotic molecules, anti-thrombotic molecules, and fibrinolytic molecules as biomarkers of lupus nephritis. Both thrombogenic and thrombolytic cascades appear to be upregulated in lupus nephritis, with proteins from both cascades appearing in the urine. Overall, urine plasmin emerged as the strongest independent predictor of eGFR and renal disease status in lupus nephritis. Whether the elevation seen in urine plasmin and TFPI in LN is the consequence of systemic or intra-renal coagulation imbalance (e.g., intra-renal thrombosis) warrants further investigation. Mechanistic studies are also warranted to test the hypothesis that elevated TFPI and plasmin may have protective roles in LN.

## Additional file

Additional file 1: **Table S1**. Levels of the urine protein markers in different disease groups. **Table S2.** Multivariate regression analysis of biomarker prediction of clinical disease. **Figure S1.** Serum Plasmin levels in different groups. (PDF 140 kb)

## Data Availability

The data generated and analyzed will be made available to interested readers.

## Abbreviations

ALN: Active LN
AUC: Area under the receiver operating characteristic curve
CKD: Chronic kidney disease
eGFR: Estimated glomerular filtration rate
FDP: Fibrin degradation products
FVIIa: Activated factor VII
GN: Glomerulonephritis
ILN: Inactive LN
LN: Lupus nephritis
ROC: Receiver operating characteristic
SLE: Systemic lupus erythematosus
SLEDAI: SLE disease activity index
SLICC RAS: The Systemic Lupus International Collaborating Clinics Renal Activity Score
TF: Tissue factor
TFPI: Tissue factor pathway inhibitor
