# Correction to: Urinary pro-thrombotic, anti-thrombotic, and fibrinolytic molecules as biomarkers of lupus nephritis

**DOI:** 10.1186/s13075-019-1966-z

**Published:** 2019-08-07

**Authors:** Ling Qin, Samantha Stanley, Huihua Ding, Ting Zhang, Van Thi Thanh Truong, Teja Celhar, Anna-Marie Fairhurst, Claudia Pedroza, Michelle Petri, Ramesh Saxena, Chandra Mohan

**Affiliations:** 1 Department of Nephrology & Rheumatology, Shanghai Tenth People’s Hospital, Tongji University School of Medicine, Shanghai, People’s Republic of China; 2 Department of Biomedical Engineering, University of Houston, 3605 Cullen Boulevard, Houston, TX 77204 USA; 3 Department of Pediatrics, UT Houston, Houston, TX USA; 4 Singapore Immunology Network, Agency for Science, Technology, and Research, Singapore, Singapore; 5 Department of Rheumatology, John Hopkins Medical University, Baltimore, MD USA; 6 Department of Nephrology, UT Southwestern Medical Center, Dallas, TX USA

**Correction to: Qin et al. Arthritis Res Ther (2019) 21:176**

**https://doi.org/10.1186/s13075-019-1959-y**

Following publication of the original article [1], it was brought to our attention that the fifth author’s name was incorrectly published. The original article [1] is corrected.

*Incorrect:*

Van Truong

*Correct:*

Van Thi Thanh Truong
