# Supplementary material for: Urinary pro-thrombotic, anti-thrombotic, and fibrinolytic molecules as biomarkers of lupus nephritis
Source: Arthritis Res Ther. 2019 Jul 18;21:176. doi: 10.1186/s13075-019-1959-y (PMC6637532; doi:10.1186/s13075-019-1959-y)

Supplementary Table 1. Levels of the urine protein markers in different disease groups.

	D-dimer , ng/mg （interquartile）	Plasmin, ug/mg （interquartile）	TF, ng/mg （interquartile）	TFPI, ng/mg （interquartile）
**ALN (n=89)**	5.159 (0, 33.32)	10.28 (2.287, 40.32)	6.431 (2.830, 14.09)	0.254 (0.1045,0.4390)
**ILN (n=24)**	1.91 (0, 4.2)	0.97 (0.49, 1.76)	2.73 (1.38, 5.76)	0.11 (0.02, 0.17)
**LN (n=113)**	3.36 (0, 24.79)	6.84 (1.18, 26.99)	5.45 (2.16, 10.88)	0.2 (0.09, 0.38)
**CKD (n=45)**	2.681 (3.408, 27.51)	2.733 (0.9656, 15.72)	4.055 (1.716, 7.492)	0.14 (0.0565, 0.245)
**HC (n=40)**	0 (0, 4.088)	0.3006 (0.1199, 0.5960)	3.769 (1.425, 10.03)	0.106 (0.038, 0.1595)
	**Fold change**	**Fold change**	**Fold change**	**Fold change**
**ALN vs ILN**	57.8*	14.6****	1.3*	1.8***
**ALN vs CKD**	4.1	1.9**	1.8**	1.6***
**ALN vs HC**	66.1***	81.5****	1.4*	3.5****

**ILN vs HC**	1.1	5.6****	1.1	1.9
**ILN vs CKD**	0.1	0.1**	1.4	0.9
**LN vs CKD**	3.2	1.6	1.7*	1.5**
**LN vs HC**	52.2***	65.2****	1.3	3.2****

Data are presented as Median (interquartile) ****p<0.0001 ***p<0.001 **p<0.01 *p<0.05

SupplementaryTable 2. Multivariate regression analysis of biomarker prediction of clinical disease

	Independent Predictors of eGFR?
D-dimer	NS
Plasmin	0.016
TF	NS
TFPI	0.027
Age	0.006
Gender	NS
Ethnicity	NS
	**Independent Predictors of SLEDAI?**
D-dimer	NS
Plasmin	0.029
TF	NS
TFPI	0.005
Age	NS
Gender	0.023
Ethnicity	NS

These urine biomarkers were independent predictors of the indicated clinical indices, after correction for age, gender and ethnicity, in multivariate regression analysis.

Supplementary Figure S1

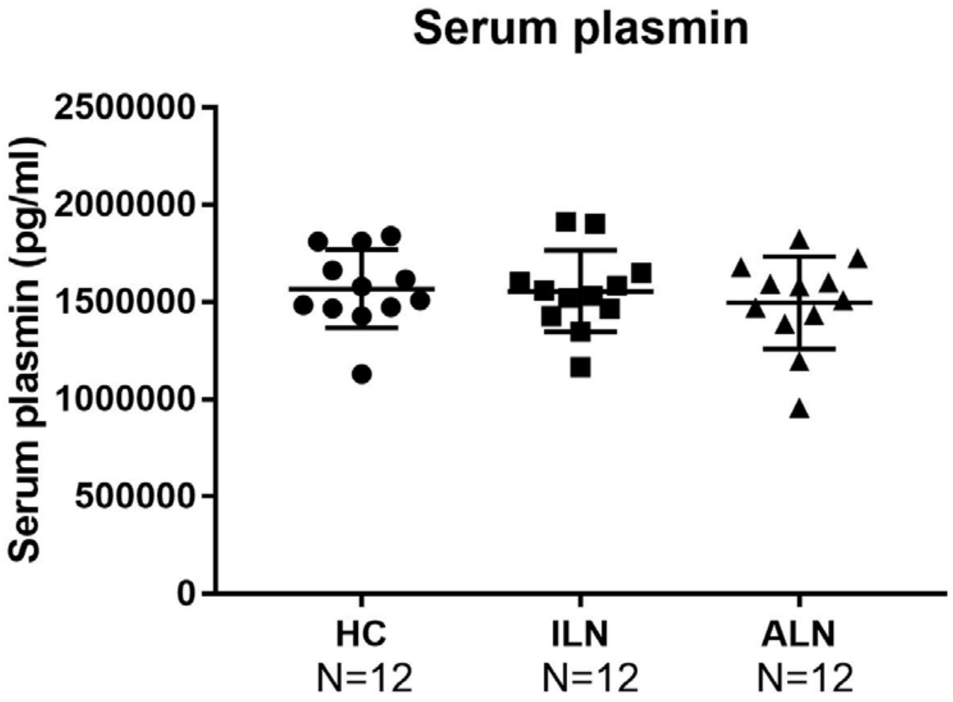

Supplement: Supplementary file 1 — Table S1. Levels of the urine protein markers in different disease groups. Table S2. Multivariate regression analysis of biomarker prediction of clinical disease. Figure S1. Serum Plasmin levels in different groups. (PDF 140 kb) [file 13075_2019_1959_MOESM1_ESM.pdf]
